# Structural Violence Education: A Critical Moment for Psychiatric Training

**DOI:** 10.1177/07067437211016243

**Published:** 2021-06-07

**Authors:** Kavya Anchuri, Natalie Jacox, Taelina Andreychuk, Allison Brown

**Affiliations:** 1 70401 University of Calgary, Cumming School of Medicine, Undergraduate Medical Education, Alberta, Canada; 2 Department of Medicine, 70401 University of Calgary, Cumming School of Medicine, Alberta, Canada; 3 Department of Community Health Sciences, 70401 University of Calgary, Cumming School of Medicine, Alberta, Canada

**Keywords:** structural violence, racism, mental health, medical education

## Abstract

The mental health ramifications of structural violence are borne disproportionately by marginalized patient populations in North America, which includes Black, Indigenous, and 2SLGBTQIA+ communities and people who use drugs. Structural violence can comprise, for example, police or state violence, colonialism, and medical violence. We chronicle the history of psychiatric discourse around structural violence over the past 50 years and highlight the critical need for new formalized competencies to become incorporated into the training of medical students across Canada, specifically addressing the impacts of structural violence for the aforementioned populations. Finally, we offer a framework of learning objectives for designing educational sessions discussing structural violence and mental health for integration into pre-clerkship psychiatry curricula at medical schools across Canada.

That the practice of medicine is inherently political—so declared by the likes of Dr. Rudolf Virchow—is perhaps no more obvious than in psychiatry and the ontologies it employs. Famed political philosopher and Black psychiatrist Dr. Frantz Fanon of French Martinique wrote decades ago of the psychopathological ramifications of colonialism on the colonized. In 1963, Fanon lamented that as “a systematic negation of the other person and a furious determination to deny [them] all attributes of humanity, colonialism forces the people it dominates to ask themselves the question constantly: ‘In reality, who am I?’ In the period of colonization […] when the sum total of harmful nervous stimuli overstep a certain threshold, […] the natives […] find themselves crowding the mental hospitals. There is thus during this calm period of successful colonization a regular and important mental pathology which is the direct product of oppression.”^1^

Nearly 60 years ago, Fanon posited systemic oppression as a precursor to mental illness. If mental health and physical circumstance are entwined by the “harmful nervous stimuli” posed by circumstance, then to teach and problematize disease and disorder without the context of circumstance not only reflects an inadequate understanding of mental illness; it is a disservice to the physicians we train and the societies they serve. To assess and treat mental illness in any community requires learning about the ongoing subjugation and structural violence which predisposes its most marginalized members to mental illness. In an era when collective social awareness of mental health is ever-expanding, several key structural determinants of mental health have been overlooked by mainstream mental health education. Namely: racism, colonialism, capitalism, classism, homophobia, transphobia, and stigma. It is in addressing these structural determinants that Michel Foucault’s proclamation becomes self-evident: “the first task of the doctor is […] political.”^2^

The colonial warfare that Fanon describes has today been replaced by newer forms of structural violence that bear consequence on the health of our communities. In its overt forms, structural violence can include state-sanctioned violence, police brutality, or trauma perpetrated by the healthcare system and healthcare professionals while practicing medicine. Its insidious forms can include neglect and systematic de-prioritization, intergenerational trauma precipitated by historical colonial violence, and impoverishment by unbridled capitalism, among other processes. Among the populations who present to psychiatric services in the developed world, structural violence disproportionately befalls persons who are Black, Indigenous, people of colour, 2SLGBTQIA+, economically disadvantaged, precariously housed or homeless, and people who use drugs—manifesting as stark inequities in health outcomes and mortality.^3–5^

Our recent sociopolitical zeitgeist of mobilization around anti-racism and dismantling systemic oppression—sparked by the murder of George Floyd by police in Minneapolis on May 25th, 2020—has catalyzed a pedagogical transformation in academic medicine. Medical leadership who were previously unattuned to the role of systemic oppression in medicine are now seeking expert guidance on its integration into core medical curricula, lest their institutions get left behind in the tide of progress. As roadmaps for this integration emerge, we draw specific attention to how psychiatrist Jonathan Metzl and colleagues examine racism and mental health, articulating the need for “structural competencies” among physicians and trainees.^6^ Evolving beyond the traditional focus on cross-cultural competencies in medicine, structural competency refers to the ability of physicians to understand how health disparities arise from larger, structural factors beyond individual control. This includes understanding how social, economic, and political systems precipitate injustices by race, class, and gender, such as inequities in wealth, education, and housing—among many other examples. This approach transcends the classical focus on social determinants of health, which identify factors and stratifications that affect disease risk and susceptibility (such as socioeconomic status, for example), to instead delineate the social, political, economic, and environmental forces that produce health inequities in the first place (such as capitalism, classism, and labour exploitation which establish and reinforce socioeconomic strata). Examples of structural competency curricula in general medical education have been recently described.^7,8^ Building on this foundation, we designed and evaluated a mandatory, interactive educational session specifically for integrating into our medical school’s pre-clerkship psychiatry curriculum, teaching about the impacts of structural violence on the mental health of marginalized communities. We delivered this training to 153 medical students at a major Canadian university. Leveraging the transition to online learning during COVID-19, we invited speakers from around Canada with collective expertise in psychiatry, clinical psychology, anti-racism, police violence, and Indigenous health, all recruited from the organizers’ academic and professional networks.

The learning objectives of this session were developed collaboratively by a group of 5 medical students who organized the session, including members of our institution’s Black Medical Students’ Association who had previously distributed a detailed roadmap on integrating anti-racist pedagogies into the medical curriculum.^9^ We drew from this roadmap to design learning objectives that were feasible for the timeframe of the session, while ensuring that they would subsume content on psychiatric effects of structural violence and the wielding of structural violence to enforce the criminalization of mental illness. We sought feedback from faculty mentors and key stakeholders throughout the development of this session, including a member of the decanal team. Feedback refined and condensed the learning objectives into pithy, digestible, and actionable statements; these learning objectives are outlined in Table 1 and are adaptable for medical schools and residency programs throughout and beyond Canada. After the session was completed, we solicited survey feedback from attendees in an effort to evaluate the operationalizability of our designed objectives and utility of such learning sessions. Qualitative evaluations of this session revealed overwhelmingly positive feedback regarding its uniqueness, pedagogical benefit, relevance and applicability to medical practice, and needed contribution to anti-racist discourse in medical education. We also asked participants to appraise the extent to which it addressed unmet needs in the medical curriculum. Participants consistently commented on the benefits of the panel, specifically the diverse perspectives it centred and the practical information gained through this session in terms of beginning to recognize the intersection of structural violence with clinical medicine and incorporating critical allyship into daily practice. An overwhelming number of learners expressed their desire for similar sessions to be integrated throughout the core curriculum.

**Table 1.** Learning Objectives.

Identify the structural, colonial, and police violence that disproportionately befalls people who are Black, Indigenous, 2SLGBTQIA+, and people who use drugs. Explain the effects of such violence on the lived experience of these communities, specifically the mental health effects: e.g. collective & community trauma, chronic stress, anxiety, and PTSD. Recognize that police are regularly called to respond to mental health crises in the community and reflect on what this implies about the criminalization of mental illness, including the criminalization of substance use disorder. In the context of your own power and positionality, reflect on how to continue learning about and mitigating the effects of structural violence throughout your medical career, thereby growing the seed of critical allyship planted by this introductory session. We understand ‘critical allyship’ in this context to involve actions, behaviours, and attitudes—both individual and systemic—that serve to explicitly reduce the burden of oppression faced by marginalized groups of which one is not a member, with the goal of advancing justice for those groups, even and especially when this requires a loss of opportunity or advancement for oneself.

*Note*: Learning objectives for an interactive educational session on structural violence and mental health among marginalized com munities, designed for medical school psychiatry curricula.

This moment is critical for academic medicine, requiring introspection on doctors’ roles in dismantling violent systems that harm the patients we vow to heal.^10^ As medicine looks outward, we must also look inward, acknowledging that our institutions are not immune to structural violence, and that this is incongruent with our primary edict, our occupational doctrine: first, do no harm. As the profession of medicine publicly condemns racism and broadly articulates commitments to racial justice, we must emphasize longitudinal structural competencies in the core medical curriculum. Failure to enact institutional-level changes in the training of physicians will allow violence and inequities to perpetuate that defy medicine’s professional oath—our social contract with society.
